# Healthcare-associated infections and antimicrobial resistance in Canadian acute care hospitals, 2017–2021

**DOI:** 10.14745/ccdr.v49i05a09

**Published:** 2023-05-01

**Authors:** Canadian Nosocomial Infection Surveillance Program

**Affiliations:** 1 Centre for Communicable Diseases and Infection Control, Public Health Agency of Canada, Ottawa, ON

**Keywords:** healthcare-associated infections, community-associated infections, antimicrobial resistance, surveillance, Clostridioides difficile infection, methicillin-resistant Staphylococcus aureus, vancomycin-resistant Enterococcus, carbapenemase-producing *Enterobacterales*, *Escherichia coli*, *Candida auris*, Canadian Nosocomial Infection Surveillance Program

## Abstract

**Background:**

Healthcare-associated infections (HAIs) and antimicrobial resistance (AMR) continue to contribute to excess morbidity and mortality among Canadians. This report describes epidemiologic and laboratory characteristics and trends of HAIs and AMR from 2017 to 2021 (*Candida auris* 2012–2021) using surveillance and laboratory data submitted by hospitals to the Canadian Nosocomial Infection Surveillance Program (CNISP) and by provincial laboratories to the National Microbiology Laboratory (NML).

**Methods:**

Data collected from 88 Canadian sentinel acute care hospitals between January 1, 2017, and December 31, 2021, for *Clostridioides difficile* infections (CDI), carbapenemase-producing *Enterobacterales* (CPE), methicillin-resistant *Staphylococcus aureus* (MRSA) bloodstream infections (BSIs) and vancomycin-resistant *Enterococcus* (VRE) BSIs. *Candida auris* (*C. auris*) surveillance was initiated in 2019 by CNISP and in 2012 by the NML. Case counts, rates, outcomes, molecular characterization and antimicrobial resistance profiles are presented.

**Results:**

From 2017 to 2021, increased rates per 10,000 patient days were observed for MRSA BSIs (35%; 0.84–1.13), VRE BSIs (43%; 0.23–0.33) and CPE infections (166%, 0.03–0.08). CDI rates decreased 11% (5.68–5.05). Thirty-one *C. auris* isolates were identified in Canada from 2012 to 2021, with the majority from Western Canada (68%).

**Conclusion:**

From 2017 to 2021, the incidence of MRSA and VRE BSIs, and CPE infections increased in Canadian acute care hospitals participating in a national sentinel network (CNISP) while CDI decreased. Few *C. auris* isolates were identified from 2012 to 2021. Reporting standardized surveillance data and the consistent application of infection prevention and control practises in acute care hospitals are critical to help decrease the burden of HAIs and AMR in Canada.

## Introduction

Healthcare-associated infections (HAIs), including antimicrobial resistant organisms, continue to place a significant burden on the Canadian healthcare system, and cause excess morbidity and mortality ((1–5)). Point-prevalence studies conducted in Canada and across Europe in 2017 have estimated 6.5%–7.9% of patients in acute care facilities had at least one HAI ((6,7)). The United States Centers for Disease Control and Prevention estimated that one in 31 hospitalized patients were infected with an HAI, corresponding to 687,000 infections and 72,000 deaths each year ((8)).

Antimicrobial resistance (AMR) threatens the treatment of HAIs, and has been identified as a global health threat by the World Health Organization ((9)). A global burden study estimated that 1.27 million deaths were attributable to bacterial AMR in 2019 ((10)). In Canada, it was estimated that 14,000 deaths were associated with AMR in 2018, with an estimated cost to the healthcare sector of $1.4 billion per year, projecting to increase to $7.6 billion per year by 2050 ((11). During the coronavirus disease 2019 (COVID-19) pandemic that was declared on March 11, 2020 ((12)), changes in hospital infection prevention and control and antimicrobial stewardship efforts had varied impacts on the rates of HAIs and AMR ((13,14)). Coordinated global public health action and improved antibiotic stewardship and public awareness are crucial to identify patterns of antimicrobial resistance and prevent and control emerging infections.

In Canada, the Public Health Agency of Canada collects national data on various HAIs and AMR through the Canadian Nosocomial Infection Surveillance Program (CNISP). Established in 1994, CNISP is a collaboration between the Public Health Agency of Canada, the Association of Medical Microbiology and Infectious Disease Canada and sentinel hospitals from across Canada. The goal of CNISP is to facilitate and inform the prevention, control and reduction of HAIs and antimicrobial resistant organisms in Canadian acute care hospitals through active surveillance and reporting.

In line with the World Health Organization’s core components of infection prevention and control ((14)), CNISP performs consistent, standardized surveillance to reliably estimate HAI burden, establish benchmark rates for national and international comparison, identify potential risk factors and assess and inform specific interventions to improve patient health outcomes. Data provided by CNISP directly supports the collaborative goals outlined in the 2017 Pan-Canadian Framework for Action for tackling AMR and antimicrobial use ((9)).

In this report, we describe the most recent HAI and AMR surveillance data collected from CNISP participating hospitals between 2017 and 2021. Further, for the first time, we provide an epidemiological summary of *Candida auris* (*C. auris*) isolates identified from 2012 to 2021 to contextualize this emerging pathogen in Canada.

## Methods

### Design

CNISP conducts prospective, sentinel surveillance for HAIs (including antimicrobial resistant organisms).

### Case definitions

Standardized case definitions for healthcare-associated (HA) and community-associated (CA) infections were used. Refer to **Appendix** for full case definitions.

### Data sources

Between January 1, 2017, and December 31, 2021, participating hospitals submitted epidemiologic data and isolates for cases meeting the respective case definitions for *Clostridioides difficile* infection (CDI), methicillin-resistant *Staphylococcus aureus* bloodstream infections (MRSA BSI), vancomycin-resistant *Enterococcus* bloodstream infections (VRE BSI) and carbapenemase-producing *Enterobacterales* (CPE) infections. Eligible *Candida auris* isolates (infections or colonizations) were identified by provincial laboratories and participating hospital laboratories between January 1, 2012, and December 31, 2021, while CNISP surveillance for *C. auris* began on January 1, 2019. In 2021, 88 hospitals in 10 provinces and one territory participated in HAI surveillance and are further described in **Table 1** and **Supplemental material, Figure S1**. In 2021, patient admissions captured in CNISP HAI surveillance were distributed across hospitals categorized as small (1–200 beds, n=38 sites, 43%), medium (201–499 beds, n=36 sites, 41%) and large (500+ beds, n=14 sites, 16%) (Table 1).

**Table 1:** Summary of hospitals participating in the Canadian Nosocomial Infection Surveillance Program, by region, 2021

Details of participating hospitals	Western^a^	Central^b^	Eastern^c^	Northern^d^	Total
Total number of hospitals	29	32	26	1	88
**Hospital type**					
Adult^e^	12	21	16	0	49
Mixed	13	7	9	1	30
Paediatric	4	4	1	0	9
**Hospital size**					
Small (1–200 beds)	11	8	18	1	38
Medium (201–499 beds)	10	18	8	0	36
Large (500+ beds)	8	6	0	0	14
**Admissions and discharge**					
Total number of beds	9,707	12,155	3,302	22	25,186
Total number of admissions	435,550	522,198	104,531	2,272	1,064,551
Total number of patient days	3,281,963	3,860,904	952,460	6,084	8,101,411

^a^ Western refers to British Columbia, Alberta, Saskatchewan and Manitoba

^b^ Central refers to Ontario and Québec

^c^ Eastern refers to Nova Scotia, New Brunswick, Prince Edward Island and Newfoundland and Labrador

^d^ Northern refers to Nunavut

^e^ Seven hospitals classified as “adult” had a neonatal intensive care unit

Epidemiologic (demographic, clinical and outcomes) and denominator data (patient days and patient admissions) were collected and submitted by participating hospitals through the Canadian Network for Public Health Intelligence—a secure online data platform.

Reviews of standardized protocols and case definitions were conducted annually by established infectious disease expert working groups; training for data submission was provided to participating CNISP hospital staff as required. Data quality for surveillance projects was periodically evaluated; methodology has been published previously ((15,16)).

### Laboratory data

Patient-linked laboratory isolates (stool samples for CDI cases) were sent to the Public Health Agency of Canada’s National Microbiology Laboratory (NML) for molecular characterization and susceptibility testing. Isolates for MRSA BSI, VRE BSI, CPE, *C. auris* (2019–2021) and paediatric CDI were submitted year-round. Adult CDI isolates were submitted annually during a targeted two-month period (March 1 to April 30). Provincial laboratories have submitted *C. auris* isolates to NML since 2012.

### Statistical analysis

Rates of HAI were calculated by dividing the total number of cases identified in patients admitted to CNISP participating hospitals by the total number of patient admissions (multiplied by 1,000) or patient days (multiplied by 10,000). The HAI rates are reported nationally and by region (Western: British Columbia, Alberta, Saskatchewan and Manitoba; Central: Ontario and Québec; Eastern: Nova Scotia, New Brunswick, Prince Edward Island and Newfoundland and Labrador; Northern: Nunavut). Sites that were unable to provide case data were excluded from rate calculations and missing denominator data were estimated using their previous years reported data, where applicable. Missing epidemiological and molecular data were excluded from analysis. The Mann-Kendall test was used to test trends. Significance testing was two-tailed and differences were considered significant at *p*≤0.05.

Where available, attributable and all-cause mortality were reported for HAIs. Attributable mortality rate was defined as the number of deaths per 100 HAI cases where the HAI was the direct cause of death or contributed to death within 30 days of positive culture or histopathology specimen, as determined by physician review. All-cause mortality rate was defined as the number of deaths per 100 HAI cases 30 days following positive culture.

## Results

### *Clostridioides difficile* infection

Between 2017 and 2021, overall CDI rates decreased by 11% (5.68 to 5.05 infections per 10,000 patient days); however, this decreasing trend was not significant (*p*=0.142) (**Table 2**).Stratified by source of infection, the incidence of HA-CDI decreased significantly; by 15.5% from 4.19–3.54 infections per 10,000 patient days (*p*=0.050) (**Table S1.1**). Community-associated-CDI (Appendix) rates remained stable when comparing 2017 to 2021 rates per 1,000 patient admissions.

**Table 2:** *Clostridioides difficile* infection data, Canada, 2017–2021^a^

*C. difficile* infection data	Year									
	2017		2018		2019		2020		2021	
**Number of infections and incidence rates**										
Number of *C. difficile* infection cases	4,018		3,850		3,600		3,654		3,572	
Rate per 1,000 patient admissions	4.29		4.15		3.70		3.97		3.94	
Rate per 10,000 patient days	5.68		5.42		4.90		5.35		5.05	
Number of reporting hospitals	68		68		73		82		80	
Attributable mortality rate per 100 cases (%)^b^	2.6		1.2		2.2		2.5		2.2	
**Antimicrobial resistance^c^**	**n**	**%**	**n**	**%**	**n**	**%**	**n**	**%**	**n**	**%**
Clindamycin	149	22.0	307	48.7	221	38.9	62	17.1	64	11.9
Moxifloxacin	114	16.9	70	11.1	66	11.6	24	6.6	49	9.1
Rifampin	14	2.1	10	1.6	6	1.1	3	0.8	9	1.7
Metronidazole	0	0.0	1	0.2	0	0.0	0	0.0	0	0.0
Total number of isolates tested^d^	676	N/A	631	N/A	568	N/A	363	N/A	538	N/A

Abbreviations: *C. difficile, Clostridioides difficile*; N/A, not applicable

^a^ All *C. difficile* isolates from 2017 to 2021 submitted to National Microbiology Laboratory were susceptible to tigecycline and vancomycin

^b^ Deaths where *C. difficile* infection was the direct cause of death or contributed to death 30 days after the date of the first positive lab specimen or positive histopathology specimen. Mortality data are collected during the two-month period (March and April of each year) for adults (age 18 years and older) and year-round for children (age one year to younger than 18 years old). Among paediatric patients, there was no death attributable to healthcare-associated *C. difficile* infection

^c^ *C. difficile* infection isolates are collected for resistance testing during the two-month period (March and April of each year) for adults (age 18 years and older) and year-round for children (age one year to younger than 18 years old) from admitted patients only

^d^ Total number reflects the number of isolates tested for each of the antibiotics listed above

Regionally, HA-CDI rates have decreased across all regions except in the East where rates have remained relatively consistent. For CA-CDI, Central region rates remain highest overall from 2017 and 2021 (range: 1.39–1.66), followed by the Western and Eastern region. Overall CDI attributable mortality remained low and fluctuated (range: 1.2–2.6 deaths per 100 cases) from 2017 to 2021 (*p*=0.801) (**Table S1.1**).

The proportion of *C. difficile* isolates resistant to moxifloxacin decreased by 7.8% between 2017 (16.9%, n=114/676) and 2021 (9.1%, n=49/538) (**Table 2**). Since 2017, moxifloxacin resistance decreased significantly among HA-CDI isolates (8.7%, *p*=0.050) while a smaller non-significant decrease was observed among CA-CDI (3.9%, *p*=0.327) (**Table S1.2**). All tested *C. difficile* isolates were susceptible to vancomycin and tigecycline. There was a single case of metronidazole resistance identified in 2018. From 2017 to 2021, the prevalence of ribotype 027 associated with NAP1 decreased for both HA and CA-CDI (by 7.7% from 15.4% to 7.7% and 4.6% from 14.7% to 11.0%, respectively) (**Table S1.3**).

### Methicillin-resistant *Staphylococcus aureus* bloodstream infections

Between 2017 and 2021, overall MRSA BSI rates increased by 35% (0.84–1.13 infections per 10,000 patient days), with a peak rate observed in 2020 (1.16 infections per 10,000 patient days) (**Table 3**). Stratified by case type, a continued steady increase (80%, *p*=0.05) was observed from 2017 to 2021 in CA-MRSA BSI rates compared to HA-MRSA BSI rates, which remained stable over time (range: 0.43–0.50 infections per 10,000 patient days) (**Table S2.1**).

**Table 3:** Methicillin-resistant *Staphylococcus aureus* bloodstream infections data, Canada, 2017–2021

MRSA BSI data	Year									
	2017		2018		2019		2020		2021	
**Number of infections and incidence rates**										
Number of MRSA bloodstream infections	606		767		888		873		855	
Rate per 1,000 patient admissions	0.61		0.78		0.85		0.86		0.84	
Rate per 10,000 patient days	0.84		1.05		1.14		1.16		1.13	
Number of reporting hospitals	65		62		69		81		78	
**All-cause mortality rate^a^**										
Number of deaths	99		144		144		152		159	
All-cause mortality rate per 100 cases	16.4		18.8		16.2		17.4		18.6	
**Antimicrobial resistance^b^**	**n**	**%**	**n**	**%**	**n**	**%**	**n**	**%**	**n**	**%**
Erythromycin	455	80.7	527	75.6	602	75.6	501	72.2	440	68.1
Ciprofloxacin	432	76.6	503	72.2	560	70.4	454	65.4	414	64.1
Clindamycin	239	42.4	287	41.2	297	37.3	229	33.0	185	28.6
Tetracycline	35	6.2	49	7.0	62	7.8	46	6.6	51	7.9
Trimethoprim/sulfamethoxazole	8	1.4	13	1.9	15	1.9	16	2.3	27	4.2
Rifampin	9	1.6	6	0.9	7	0.9	6	0.9	8	1.2
Tigecycline	0	0	0	0	0	0	1	0.1	2	0.3
Daptomycin	5	0.9	0	0	3	0.4	5	0.7	5	0.8
Total number of isolates tested^c,d^	564	N/A	697	N/A	796	N/A	694	N/A	646	N/A

Abbreviations: MRSA, methicillin-resistant *Staphylococcus aureus*; MRSA BSI, methicillin-resistant *Staphylococcus aureus* bloodstream infection; N/A, not applicable

^a^ Based on the number of cases with associated 30-day outcome data

^b^ All MRSA isolates from 2017 to 2021 submitted to National Microbiology Laboratory were susceptible to linezolid and vancomycin

^c^ In some years, the number of isolates tested for resistance varied by antibiotic

^d^ Total number reflects the number of isolates tested for each of the antibiotics listed above

In 2021, HA-MRSA BSI and CA-MRSA BSI rates were highest in Western Canada (0.47 and 0.82 infections per 10,000 patient days, respectively) (Table S2.1). Among hospital types, HA and CA-MRSA BSI rates have generally remained highest among adult and mixed hospitals. Stratified by hospital size, rates of HA-MRSA BSI were highest among medium (201–499 beds) and large size hospitals (500+ beds) while CA-MRSA BSI rates have been highest in medium size hospitals since 2019. All-cause mortality remained relatively stable from 2017 to 2021 (range: 16.2%–18.8%) (Table 3). In 2021, 30-day all-cause mortality was higher among those with HA-MRSA (24.8%) compared to those with CA-MRSA (15.0%).

Clindamycin resistance among MRSA isolates decreased significantly by 13.8% between 2017 (42.4%, n=239/564) and 2021 (28.6%, n=185/646) (*p*=0.0143) (Table 3). Since 2017, the proportion of MRSA isolates with erythromycin and ciprofloxacin resistance decreased, yet remained high (68.1% and 64.1% in 2021, respectively) in relation to other antibiotics tested. Between 2017 and 2021, daptomycin non-susceptibility was detected in 18 isolates. All submitted MRSA BSI isolates from 2017 to 2021 were susceptible to linezolid and vancomycin.

Comparing HA-MRSA isolates to CA-MRSA isolates, clindamycin resistance was consistently higher among HA-MRSA isolates each year from 2017 (47.3% vs. 36.6%) to 2021 (36.3% vs. 23.9%) (**Table S2.2**). There were no other notable differences in antibiotic resistance patterns by MRSA BSI case type.

Between 2017 and 2021, the proportion of spa types identified as t002 (CMRSA2) and most commonly associated with MRSA infections acquired in a healthcare setting continued to decrease; from 23.5% of all HA-MRSA isolates in 2017 to 15.6% in 2021. The proportion of spa types identified as t008 (CMRSA10) and most commonly associated with MRSA infections acquired in the community continued to increase and account for the largest proportion of CA-MRSA isolates from 2017 (45.3%) to 2021 (48.9%) (**Table S2.3**).

### Vancomycin-resistant *Enterococcus* bloodstream infections

From 2017 to 2018, VRE BSI rates increased by 43%, from 0.23 to 0.33 infections per 10,000 patient days while rates remained elevated but stable from 2018 to 2021 (range: 0.30–0.33 infections per 10,000 patient days) (**Table 4**). Regionally, VRE BSI rates were highest in Western and Central Canada (0.42 and 0.34 infections per 10,000 patient days in 2021, respectively) with few VRE BSIs reported in Eastern Canada (range: 0–0.02 infections per 10,000 patient days) (**Table S3.1**). Stratified by hospital type, VRE BSI rates remained highest in adult hospitals from 2017 to 2021 (range: 0.29–0.45 infections per 10,000 patient days). From 2017 to 2021, VRE BSI rates in paediatric hospitals were low, with zero cases reported in 2021. In 2021, VRE BSI rates were 0.36 infections per 10,000 patient days in both medium (201–499 beds) and large (500+ beds) size hospitals while rates in small (1–200 beds) hospitals have decreased since 2019 (0.35 to 0.14 infections per 10,000 patient days).

**Table 4:** Vancomycin-resistant *Enterococcus faecium* bloodstream infections data, 2017–2021

VRE BSI data	Year									
	2017		2018		2019		2020		2021	
**Vancomycin-resistant *Enterococcus* bloodstream infections data**										
Number of VRE BSIs	154		242		241		223		246	
Rate per 1,000 patient admissions	0.16		0.25		0.23		0.22		0.25	
Rate per 10,000 patient days	0.23		0.33		0.30		0.30		0.33	
Number of reporting hospitals	59		62		70		80		76	
**Antimicrobial resistance of *Enterococcus faecium* isolates**	**n**	**%**	**n**	**%**	**n**	**%**	**n**	**%**	**n**	**%**
Ampicillin	115	100	180	100	173	100	130	98.5	142	99.3
Chloramphenicol	11	9.6	4	2.2	30	17.3	28	21.2	48	33.6
Ciprofloxacin	115	100	180	100	173	100	131	99.2	142	99.3
Daptomycin^a^	9	7.8	11	6.1	7	4.0	4	3.0	2	1.4
Erythromycin	107	93.0	172	95.6	166	96.0	126	95.5	135	94.4
High-level gentamicin	44	38.3	76	42.2	57	32.9	35	26.5	26	18.2
Levofloxacin	115	100	178	98.9	173	100	130	98.5	142	99.3
Linezolid	0	0.0	2	1.1	3	1.7	1	0.8	1	0.7
Nitrofurantoin	51	44.3	54	30.0	66	38.2	54	40.9	112	78.3
Penicillin	115	100	180	100	173	100	131	99.2	142	99.3
Quinupristin/dalfopristin	8	7.0	18	10.0	18	10.4	7	5.3	4	2.8
Rifampicin	109	94.8	162	90.0	160	92.5	114	86.4	131	91.6
High-level streptomycin	39	33.9	60	33.3	42	24.3	29	22.0	39	27.3
Tetracycline	65	56.5	107	59.4	119	68.8	88	66.7	114	79.7
Tigecycline	0	0.0	1	0.6	0	0.0	0	0.0	0	0.0
Vancomycin	110	95.7	175	97.2	170	98.3	128	97.0	138	96.5
Total number of isolates tested^b^	115	N/A	180	N/A	173	N/A	132	N/A	143	N/A

Abbreviations: N/A, not applicable; VRE BSI, vancomycin-resistant *Enterococcus* bloodstream infection

^a^ Clinical and Laboratory Standards Institute (CLSI) resistance breakpoints came into effect in 2019 and was applied to all years

^b^ Total number reflects the number of isolates tested for each of the antibiotics listed above

Note: Aggregate mortality data reported in-text due to fluctuations in the small numbers of VRE BSI deaths reported each year

Vancomycin-resistant *Enterococcus* BSI were predominantly HA, as 89.9% (n=994/1,106) of VRE BSI reported from 2017 to 2021 were acquired in a healthcare facility. All-cause mortality remained high (32.6%) from 2017 to 2021. The incidence rates by region, hospital type and hospital size are presented in **Table S3.2**.

Between 2017 to 2021, high-level gentamicin resistance among VRE BSI isolates (*Enterococcus faecium*) decreased from 38.3% to 18.2% (*p*=0.05) (Table 4). Daptomycin non-susceptibility, first identified in 2016, has decreased from 7.8% (n=9 isolates) in 2017 to 1.4% (n=2 isolates) in 2021 (*p*=0.0143). Since 2017, the majority (99.4%) of VRE BSI isolates were identified as *Enterococcus faecium*; however, three *E. faecalis* were identified in 2018 and one in 2020 (**Table S3.3**). Among *E. faecium* isolates, the proportion identified as sequence type (ST)1478 was highest in 2018 (37.2%, n=67/180) and decreased to 7.0% (n=10/143) in 2021 (*p*=0.0415) (**Table S3.4**). Furthermore, the proportion of ST17 isolates significantly increased from 2017 (6.1%, n=7/115) to 2021 (53.8%, n=77/143) (*p*=0.05) (Table S3.4).

### Carbapenemase-producing *Enterobacterales*

From 2017 to 2021, CPE infection rates have remained low. A slight increase was observed from 2017 to 2018 (0.03 to 0.06 infections per 10,000 patient days, respectively) and rates have remained stable from 2018 to 2021 (**Table 5**).

**Table 5:** Carbapenemase-producing *Enterobacterales* data, Canada, 2017–2021^a^

CPE data	Year													
	2017		2018		2019				2020			2021		
**Number of infections and incidence rates**														
Number of CPE infections	20		36		50				41			55		
Infection rate per 1,000 patient admissions	0.02		0.04		0.06				0.05			0.06		
Infection rate per 1,000 patient days	0.3		0.6		0.8				0.6			0.8		
Infection rate per 10,000 patient days	0.03		0.06		0.08				0.06			0.08		
Number of reporting hospitals	52		51		59				75			77		
**Drugs tested for antimicrobial resistance**														
**Antibiotics^b,c^**	**n**	**%**	**n**	**%**		**n**		**%**	**n**	**%**	**n**		**%**	
Piperacillin-Tazobactam	159	85.0	210	92.1		237		90.8	230	93.9	262		92.3	
Ceftriaxone	173	92.5	212	93.0		250		95.8	218	88.9	244		85.9	
Ceftazidime	160	85.6	192	84.2		233		89.3	203	82.9	225		79.2	
Meropenem	159	85.0	198	86.8		190		72.8	149	60.8	183		64.4	
Ciprofloxacin	138	73.8	158	69.3		183		70.1	173	70.6	195		68.7	
Amikacin	32	17.1	44	19.3		23		8.8	24	9.8	22		7.7	
Gentamicin	64	34.2	80	35.1		86		33.0	76	31	78		27.5	
Tobramycin	71	38.0	101	44.3		121		46.4	91	37.1	106		37.2	
Trimethoprim-sulfamethoxazole	113	60.4	143	62.7		193		73.9	184	75.1	204		71.8	
Tigecycline	18	9.6	30	13.2		36		13.8	0	0	1		0.4	
Total number of isolates tested^d^	187	N/A	228	N/A		261		N/A	245	N/A	284		N/A	
**Carbapenemases identified**														
KPC	86	46.0	122	53.0		127	48.5		98	40		133		46.8
NDM	53	28.3	59	25.7		74	28.2		80	32.7		74		26.1
OXA-48	33	17.6	30	13.0		40	15.3		48	19.6		45		15.8
SME^e^	2	1.1	4	1.7		1	0.4		2	0.8		1		0.4
NDM/OXA-48	5	2.7	6	2.6		10	3.8		9	3.7		11		3.9
GES	1	0.5	1	0.4		2	0.8		0	0		1		0.4
IMP	0	0.0	3	1.3		1	0.4		1	0.4		1		0.4
NMC	4	2.1	2	0.9		4	1.5		7	2.9		15		5.3
VIM	3	1.6	3	1.3		3	1.1		0	0		1		0.4
Other	0	0.0	0	0.0		0	0.0		0	0		2		0.7
Total number of isolates tested^f^	187	N/A	230	N/A		262	N/A		245	N/A		284		N/A

Abbreviations: CPE, carbapenemase-producing *Enterobacterales*; GES, Guiana extended-spectrum ß-lactamase; IMP, active-on-imipenem; KPC, *Klebsiella pneumoniae* carbapenemase; NDM, New Delhi metallo-ß-lactamase; NMC, not metalloenzyme carbapenemase; N/A, not applicable; OXA-48, Oxacillinase-48; SME, *Serratia marcescens* enzymes; VIM, Verona integron-encoded metallo-ß-lactamase

^a^ Includes data for all CPE isolates submitted

^b^ All isolates were resistant to ampicillin, and all but one to cefazolin. All carbapenemase-producing organism isolates were screened for the *mcr*-type gene which is an acquired gene associated with colistin resistance

^c^ The denominator for some drugs were adjusted as minimum inhibitory concentration values were not given in all cases due to VITEK^®^ algorithms

^d^ Total number reflects the number of isolates tested for each of the antibiotics listed above

^e^ Only found in *Serratia marcescens*

^f^ Some isolates contain multiple carbapenemases therefore the total number of isolates tested and the number of carbapenemases indicated may not match

Note: Aggregate mortality data reported in-text due to fluctuations in the small numbers of CPE deaths reported each year

From 2017 to 2021, the majority of CPE infections (97.5%) were identified in Central (50.0%, n=101/202) and Western Canada (47.0%, n=95/202) while few infections were identified in the East (3.0%; n=6/202) (**Table S4**). From 2017 to 2021, large hospitals (500+ beds) generally reported the highest rates of CPE infections (0.05–0.12 infections per 10,000 patient days). Thirty days all-cause mortality was 19.7% (n=38/193). From 2017 to 2021, 28.9% (n=48/166) of CPE infected patients reported travel outside of Canada and of those, 91.5% (n=43/47) received medical care while abroad.

From 2017 to 2021, the prevalence of amikacin and gentamicin resistance among CPE isolates decreased by 9.4% and 6.7%, respectively, while trimethoprim-sulfamethoxazole resistance increased by 11.4% (Table 5). The predominant carbapenemases identified in Canada were *Klebsiella pneumoniae* carbapenemase (KPC), New Delhi metallo-ß-lactamase (NDM) and Oxacillinase-48 (OXA-48), accounting for 88.7% of identified carbapenemases in 2021. Among submitted isolates, the proportion of carbapenemase-producing pathogens identified as *Escherichia coli* has decreased 7.1% since 2019; however, they remain the most commonly identified pathogen from 2017 to 2021 (range: 23.1%–33.7%) (**Table S5**). From 2017 to 2021, carbapenemase-producing pathogens identified as *Klebsiella pneumoniae* decreased by 7.7% while *Citrobacter freundii* increased by 9%.

### Candida auris

A total of 31 isolates (colonizations and infections) have been reported to NML from 2012 to 2021. Twenty-one cases were from Western Canada, nine cases were from Central Canada and one case was reported from Eastern Canada. Approximately, one third of isolates were resistant to amphotericin B (38.7%, n=12/31) and two thirds were resistant to fluconazole (58.1%, n=18/31). One third of isolates were multidrug-resistant (resistant to two classes of antifungals) (38.7%, n=12/31). Of the eight patients with travel information, two reported no travel (25%) while six reported international travel (75%). Of the six patients with reported history of travel, five had received healthcare abroad (83%). Of the six patients with reported travel, four had known carbapenemase-producing organism status and three were positive.

## Discussion

CNISP surveillance data have shown that between 2017 and 2021 there was a decreasing trend for CDI infection rates (including both HA and CA-cases) in Canada, but rates of MRSA and VRE BSI increased by 35% and 43%, respectively. Rates of CPE infection increased, but remained stable from 2018 to 2021 and few *C. auris* isolates were identified from 2012 to 2021. The COVID-19 pandemic has had a varied effect on the rates of HAIs in Canada and in the United States ((13,17)). Modelling HAI rates before and during the COVID-19 pandemic showed evidence of an immediate increase in HA rates of CDI while MRSA BSI, CPE and VRE BSI rates immediately decreased; however, COVID-19 pandemic status was not associated with lasting impacts on monthly rate trends in these infections ((18)). Studies have suggested pandemic-related factors that may have contributed to the changes in observed rates of HAIs, such as public health measures implemented in both the hospital and the community, population travel and mobility, changes in infection control practises, screening, laboratory testing and antimicrobial stewardship ((14)).

Declining CDI rate trends observed in the CNISP network are like those reported globally; however, rates have been reported to be higher in North America than other regions ((19)). The overall reduction in CDI rates across Canada suggests improvements in infection prevention and control practises and quality-improvement initiatives such as hand hygiene compliance, environmental cleaning, improved laboratory diagnostic techniques and antibiotic stewardship ((20,21)). In 2020, during the COVID-19 pandemic, there was evidence of an immediate increase in rates of CDI in the CNISP network, in contrast with the United States where rates continued to decline ((17)); however, the COVID-19 pandemic was not associated with a lasting impact on CDI rate trends.

In Canada, ribotype 027 continued to decrease in prevalence from 2017 to 2021, and coincided with a 7.8% decrease in moxifloxacin resistance during this time period. Furthermore, moxifloxacin resistance remained lower (9.1% in 2021) than previously published weighted pooled resistance data for North America (44.0%) and Asia (33.0%) ((22,23)). The decline in RT027 prevalence from 2017 to 2021 may also have influenced the decline in CDI rates among CNISP hospitals as this ribotype has been associated with increased virulence and fluoroquinolone resistance ((24)).

From 2017 to 2021, MRSA BSI rates continued to increase in the CNISP network, and is attributed to the increase in CA cases. Methicillin-resistant *S. aureus* BSI is associated with increased morbidity and mortality, increased length of hospital stays and increased HA costs among admitted patients ((25–28)). The 13.8% decrease in clindamycin resistance among MRSA BSI isolates from 2017 to 2021 was likely associated with the decrease in the proportion of spa type t002 (CMRSA2 epidemic type) identified among tested isolates ((29)). Healthcare-associated-MRSA BSI rates observed in the CNISP network from 2017 to 2020 (range: 0.43–0.50 infections per 10,000 patient days) were lower compared to those reported in Australian public hospitals (range: 0.71–0.76 infections per 10,000 patient days) ((30)). Based on available data in 2017 and 2018, HA-MRSA BSI rates were higher in the United States (0.52 infections per 10,000 patient days) compared to Canada (0.43–0.45 infections per 10,000 patient days) ((31)).

The increasing number of patients identified with CA-MRSA who were admitted to hospital in the CNISP network may be associated with a growing CA-MRSA reservoir, both in Canada and globally ((32,33)). Increased rates of CA-MRSA BSI suggests that strategies that target the reduction and prevention of MRSA infections in the community, especially in populations with increased risk of contracting CA-MRSA (i.e. children, athletes, incarcerated populations, people who inject drugs), such as screening and eradication of the carriage of MRSA, may be effective in reducing the burden of MRSA BSI overall ((34,35)).

The increase in VRE BSI rates in Canadian acute care hospitals is concerning as vancomycin resistance related to this infection has been shown to be a principal predictor of mortality, and is associated with increased hospital burden ((36–38)). The increase in VRE BSI rates observed in the CNISP network may be linked to changes in infection control policies, including the discontinuation of VRE screening and isolation programs in some Canadian acute care hospitals ((39)). The ST17 sequence type has contributed to the increased burden of VRE BSI in CNISP-participating hospitals by emerging as the predominant clone, overtaking ST1478. The ST17 sequence type is a globally disseminated VRE clone endemic in many countries but previously observed in low numbers in Canada ((40)). Changes in the resistance profiles of VRE BSI coincide with changes in ST distributions. The ST17 sequence type is associated with nitrofurantoin and chloramphenicol resistance, and the increase in ST17 prevalence corresponds to the increasing trend in resistance detected for these antimicrobials while daptomycin and high-level gentamicin resistance, associated with ST1478, have decreased since 2017. Vancomycin-resistant *Enterococcus* BSI trends are further impacted by the number of high-risk patients admitted to hospital (e.g. bone marrow transplants, solid organ transplants, cancer patients, etc.) ((41)). Although there is a lack of recent data on VRE BSI rates in comparable jurisdictions, there have been increasing trends noted in Europe ((42–45)), which may be associated, in part, with the introduction and spread of a new clone and gaps in infection prevention practises ((44–46)).

Carbapenemase-producing *Enterobacterales* infections are a significant threat to public health due to their resistance to last line antimicrobials, limiting treatment options for patients with an infection due to pathogens that have the propensity to rapidly spread in healthcare settings ((47–51)). While the number of CPE infections increased from 2017 to 2021 in the CNISP network, incidence remained stable from 2018 to 2021. Data on the incidence of CPE infections in other countries, such as the United Kingdom, have noted increasing incidence of CPE infections ((52,53)). Similarly, the number of CPE isolates identified through laboratory surveillance associated with CPE infections has increased in Switzerland from 2013 to 2018 ((54)). Strict implementation of infection control measures, including screening for patient travel history, is essential to reduce the transmission of CPE in Canadian acute care hospitals.

*Candida auris* is an emerging multi-drug resistant fungus which has been detected across multiple countries and continents including Canada, since its first detection in 2009. *Candida auris* has been associated with outbreaks in healthcare settings in many countries, including Canada and the United States ((55–58)), and can cause both superficial and invasive infections with mortality ranging from 30%–60% ((59)). Though still relatively rare in Canada, the United States reported almost 8,000 clinical and screening cases in a recent one-year period ((60)). We evaluated *C. auris* preparedness within CNISP hospitals in 2018 and found that most hospitals did not yet have laboratory protocols or infection prevention and control policies in place for detecting and controlling *C. auris* ((61)). The identification of *C. auris* in routine microbiology laboratories requires identification of *Candida* to the species level, which may not be routinely performed due to challenges in balancing cost with value added for clinical decision-making. Treatment options are limited for patients as one third of identified *C. auris* isolates in Canada were multi-drug-resistant and additional resistance can develop during antifungal therapy ((62)). Therefore, rapid identification, screening for colonization in at-risk patients and strict implementation of infection prevention and control measures are required to reduce the transmission of *C. auris* in Canadian healthcare settings. Continued reporting on *C. auris* in Canada is important to assess and monitor risk of this pathogen, in addition to identifying epidemiological and microbiological trends ((63)).

## Strengths and limitations

The main strength of CNISP is the collection of standardized and detailed epidemiological and laboratory-linked data from 88 sentinel hospitals across Canada for the purpose of providing national HAI and AMR trends for benchmarking and to inform hospital infection prevention and control practises. It is important to note that data in this report include those from the early years of the COVID-19 pandemic. Therefore, rates of HAIs and AMR in 2020 and 2021 may be impacted by changes in national, regional and municipal hospital-based infection prevention and control measures.

Epidemiological data collected by CNISP were limited to information available in patient charts. Hospital staff turnover may affect the consistent application of CNISP definitions when reviewing medical charts; however, these data were collected by experienced and trained infection prevention and control staff who receive periodic training with respect to CNISP methods and definitions. Furthermore, data quality assessments were conducted to maintain and improve data quality. Recruitment efforts have increased representation and coverage of Canadian acute care beds in the CNISP network from 32% to 35% from 2017 to 2021, notably among northern, rural communities and Indigenous populations.

### Next steps

Recruitment of Canadian acute care hospitals to the CNISP network in all ten provinces and three territories is an ongoing effort to improve the quality and representativeness of Canadian HAI surveillance data. Furthermore, the enhanced hospital screening practices survey is conducted annually to better understand and contextualize changes in HAI rates in the CNISP network. To further improve representativeness and generalizability of national HAI benchmark rates, CNISP and Association of Medical Microbiology and Infectious Disease Canada have launched a simplified dataset accessible to all acute care hospitals across Canada to collect and visualize annual HAI rate data. In recent years, CNISP has implemented surveillance for new and emerging pathogens, including *C. auris* and COVID-19. Studies are ongoing to assess the impact of the COVID-19 pandemic on HAI rates and AMR.

## Conclusion

Surveillance findings from a national sentinel network of Canadian acute care hospitals indicate that rates of MRSA BSI, VRE BSI and CPE infections have increased from 2017 to 2021 while rates of CDI have decreased. Few cases of *C. auris* were detected in Canada from 2012 to 2021. Consistent and standardized surveillance of epidemiologic and laboratory HAI data are essential to providing hospital practitioners with benchmark rates and informing infection prevention and control and antimicrobial stewardship policies to help reduce the burden of HAI and the impact of AMR in Canadian acute care hospitals.

## Supplemental material

These documents can be accessed on the Supplemental material file.

Figure S1: Number and proportion of patient admissions included in the Canadian Nosocomial Infection Surveillance Program by hospital type and size, 2021

Table S1.1: Cases and incidence rates of healthcare-associated and community-associated *Clostridioides difficile* infection by region, hospital type and hospital size, Canada, 2017–2021

Table S1.2: Antimicrobial resistance of healthcare-associated and community-associated *Clostridioides difficile* infection isolates, Canada, 2017–2021

Table S1.3: Number and proportion of common ribotypes of healthcare-associated and community-associated *Clostridioides difficile* infection cases, Canada, 2017–2021

Table S2.1: Cases and incidence rates of healthcare-associated and community-associated methicillin-resistant *Staphylococcus aureus* bloodstream infections by region, hospital type and hospital size, 2017–2021

Table S2.2: Antimicrobial resistance of healthcare-associated and community-associated methicillin-resistant *Staphylococcus aureus* bloodstream infection isolates, Canada, 2017–2021

Table S2.3: Number and proportion of select methicillin-resistant *Staphylococcus aureus* spa types (with corresponding epidemic types) identified

Table S3.1: Number of vancomycin-resistant *Enterococcus* bloodstream infections incidence rates by region, hospital type and hospital size, 2017–2021

Table S3.2: Number of healthcare-associated vancomycin-resistant *Enterococcus* bloodstream infections and incidence rates by region, hospital type and hospital size, 2017–2021

Table S3.3: Number and proportion of vancomycin-resistant *Enterococcus* bloodstream infections isolate types identified, 2017–2021

Table S3.4: Distribution of vancomycin-resistant *Enterococcus faecium* bloodstream sequence types, 2017–2021

Table S4: Number of carbapenemase-producing *Enterobacterales* infections and incidence rates by region, hospital type and hospital size, 2017–2021

Table S5: Number and proportion of main carbapenemase-producing pathogens identified

References

## Appendix Surveillance case definitions and eligibility criteria, 2021

### *Clostridioides difficile* infection

A “primary” episode of *Clostridioides difficile* infection (CDI) is defined either as the first episode of CDI ever experienced by the patient or a new episode of CDI that occurs greater than eight weeks after the diagnosis of a previous episode in the same patient.

A patient is identified as having CDI if:

· The patient has diarrhea or fever, abdominal pain and/or ileus AND a laboratory confirmation of a positive toxin assay or positive polymerase chain reaction (PCR) for *C. difficile* (without reasonable evidence of another cause of diarrhea)

OR

· The patient has a diagnosis of pseudomembranes on sigmoidoscopy or colonoscopy (or after colectomy) or histological/pathological diagnosis of CDI

OR

· The patient is diagnosed with toxic megacolon (in adult patients only)

Diarrhea is defined as one of the following:

· More watery/unformed stools in a 36-hour period

OR

· More watery/unformed stools in a 24-hour period and this is new or unusual for the patient (in adult patients only)

Exclusion:

• Any patients younger than one year

• Any paediatric patients (aged one year to younger than 18 years) with alternate cause of diarrhea found (i.e. rotavirus, norovirus, enema or medication, etc.) are excluded even if *C. difficile* diagnostic test result is positive

CDI case classification:

Once a patient has been identified with CDI, the infection will be classified further based on the following criteria and the best clinical judgment of the healthcare and/or infection prevention and control practitioner.

Healthcare-associated (acquired in your facility) CDI case definition:

• Related to the current hospitalization:

o The patient’s CDI symptoms occur in your healthcare facility three or more days (or 72 hours or longer) after admission

• Related to a previous hospitalization:

o Inpatient: the patient’s CDI symptoms occur less than three days after the current admission (or fewer than 72 hours) AND the patient had been previously hospitalized at your healthcare facility and discharged within the previous four weeks

o Outpatient: the patient presents with CDI symptoms at your emergency room (ER) or outpatient location AND the patient had been previously hospitalized at your healthcare facility and discharged within the previous four weeks

· Related to a previous healthcare exposure at your facility:

o Inpatient: the patient’s CDI symptoms occur less than three days after the current admission (or fewer than 72 hours) AND the patient had a previous healthcare exposure at your facility within the previous four weeks

o Outpatient: the patient presents with CDI symptoms at your ER or outpatient location AND the patient had a previous healthcare exposure at your facility within the previous four weeks

Healthcare-associated (acquired in any other healthcare facility) CDI case definition:

· Related to a previous hospitalization at any other healthcare facility:

o Inpatient: the patient’s CDI symptoms occur less than three days after the current admission (or fewer than 72 hours) AND the patient is known to have been previously hospitalized at any other healthcare facility and discharged/transferred within the previous four weeks

o Outpatient: the patient presents with of CDI symptoms at your ER or outpatient location AND the patient is known to have been previously hospitalized at any other healthcare facility and discharged/transferred within the previous four weeks

· Related to a previous healthcare exposure at any other healthcare facility:

o Inpatient: the patient’s CDI symptoms occur less than three days after the current admission (or fewer than 72 hours) AND the patient is known to have a previous healthcare exposure at any other healthcare facility within the previous four weeks

o Outpatient: the patient presents with CDI symptoms at your ER or outpatient location AND the patient is known to have a previous healthcare exposure at any other healthcare facility within the previous four weeks

Healthcare-associated CDI but unable to determine which facility:

The patient with CDI DOES meet both definitions of healthcare-associated (acquired in your facility) and healthcare-associated (acquired in any other healthcare facility), but unable to determine to which facility the case is primarily attributable to.

Community-associated CDI case definition:

· Inpatient: the patient’s CDI symptoms occur less than three days (or fewer than 72 hours) after admission, with no history of hospitalization or any other healthcare exposure within the previous 12 weeks

· Outpatient: the patient presents with CDI symptoms at your ER or outpatient location with no history of hospitalization or any other healthcare exposure within the previous 12 weeks

Indeterminate CDI case definition:

The patient with CDI does NOT meet any of the definitions listed above for healthcare-associated or community-associated CDI. The symptom onset was more than four weeks but fewer than 12 weeks after the patient was discharged from any healthcare facility or after the patient had any other healthcare exposure.

Methicillin-resistant *Staphylococcus aureus* (MRSA) infection

MRSA bloodstream infection (BSI) case definition:

· Isolation of *Staphylococcus aureus* from blood

AND

· Patient must be admitted to the hospital

AND

· Is a "newly identified *S. aureus* infection" at a Canadian Nosocomial Infection Surveillance Program (CNISP) hospital at the time of hospital admission or identified during hospitalization

### Infection inclusion criteria:

· Methicillin-susceptible *Staphylococcus aureus* (MSSA) or MRSA BSIs identified for the first time during this current hospital admission

· MSSA or MRSA BSIs that have already been identified at your site or another CNISP site but are **new** infections

Criteria to determine NEW MSSA or MRSA BSI:

· Once the patient has been identified with a MSSA or MRSA BSI, they will be classified as a new MSSA or MRSA if they meet the following criteria: more than 14 days since previously treated MSSA or MRSA BSI and in the judgment of infection control physicians and practitioners represents a new infection

### Infection exclusion criteria:

· Emergency, clinic, or other outpatient cases who are **NOT admitted** to the hospital

Healthcare-associated (HA) case definition:

Healthcare-associated is defined as an inpatient who meets the following criteria and in accordance with the best clinical judgment of the healthcare and/or infection prevention and control practitioner:

· Patient is on or beyond calendar day 3 of their hospitalization (calendar day 1 is the day of hospital admission)

OR

· Has been hospitalized in your facility in the last 7 days or up to 90 days depending on the source of the infection

OR

· Has had a healthcare exposure at your facility that would have resulted in this bacteremia (using best clinical judgment)

OR

· Any patient who has a bacteremia not acquired at your facility that is thought to be associated with any other healthcare exposure (e.g. another acute-care facility, long-term care, rehabilitation facility, clinic or exposure to a medical device)

Healthcare-associated (HA) case definition (newborn):

· The newborn is on or beyond calendar day 3 of their hospitalization (calendar day 1 is the day of hospital admission)

· The mother was **NOT** known to have MRSA on admission and there is no epidemiological reason to suspect that the mother was colonized prior to admission, even if the newborn is fewer than 48 hours of age

· In the case of a newborn transferred from another institution, MSSA or MRSA BSI may be classified as HA your acute-care facility if the organism was **NOT** known to be present and there is no epidemiological reason to suspect that acquisition occurred prior to transfer

Community-associated case definition:

· No exposure to healthcare that would have resulted in this bacteremia (using best clinical judgment) and does not meet the criteria for a healthcare-associated BSI

### Vancomycin-resistant *Enterococcus* (VRE) infection

VRE BSI case definition:

· Isolation of *Enterococcus faecalis* or *faecium* from blood

AND

· Vancomycin MIC **at least** 8 µg/ml

AND

· Patient must be admitted to the hospital

AND

· Is a "newly” identified VRE BSI at a CNISP facility at the time of hospital admission or identified during hospitalization

**A newly identified VRE BSI** is defined as a positive VRE blood isolate more than 14 days after completion of therapy for a previous infection and felt to be unrelated to previous infection in accordance with best clinical judgment by Infection Control physicians and practitioners.

Exclusion criteria:

· Emergency, clinic, or other outpatient cases who are **not admitted** to the hospital

Healthcare-associated (HA) case definition:

Healthcare-associated is defined as an inpatient who meets the following criteria and in accordance with the best clinical judgment of the healthcare and/or infection prevention and control practitioner:

· Patient is on or beyond calendar day 3 of their hospitalization (calendar day 1 is the day of hospital admission)

OR

· Has been hospitalized in your facility in the last 7 days or up to 90 days depending on the source of the infection

OR

· Has had a healthcare exposure at your facility that would have resulted in this bacteremia (using best clinical judgment)

OR

· Any patient who has a bacteremia not acquired at your facility that is thought to be associated with any other healthcare exposure (e.g. another acute-care facility, long-term care, rehabilitation facility, clinic or exposure to a medical device)

### Carbapenemase-producing *Enterobacterales* (CPE) infection

Case eligibility:

· Patient is admitted to a CNISP hospital or presents to a CNISP hospital emergency department or a CNISP hospital-based outpatient clinic

· Laboratory confirmation of carbapenem resistance or carbapenemase production in *Enterobacterales* spp.

Following molecular testing, only isolates determined to be harbouring a carbapenemase are included in surveillance. If multiple isolates are submitted for the same patient in the same surveillance year, only the isolate from the most invasive site is included in epidemiological results (e.g. rates and outcome data). However, antimicrobial susceptibility testing results represent all CPE isolates (including clinical and screening isolates from inpatients and outpatients) submitted between 2016 and 2020; duplicates (i.e. isolates from the same patient where the organism and the carbapenemase were the same) were excluded.

### Candida auris

Patients admitted to a participating hospital or presenting to a hospital emergency department or a hospital-based outpatient clinic with laboratory confirmation of *C. auris* from any specimen.

Included in this surveillance project are all clinical or screening samples that were positive for *C. auris* by any method. Currently, *C. auris* can be identified by rRNA sequencing, Vitek MS MALDI-TOF (with either the clinical database v3.2 or later or the RUO database), or Bruker MALDI-TOF (with either the clinical database v6903 or later or the RUO database). The project also includes potential *C. auris* misidentifications or “No identification” as outlined in the **Table A1** below.

**Table A1:** Laboratory identification of *Candida auris*

Identification method	Identification of suspect isolates
Vitek MS MALDI Clinical database older than v3.2	*C. haemulonii* No ID/low discrimination *C. rugosa* (not a problem for v3.0 or later) *C. pulcherrima* (not a problem for v3.0 or later)
Bruker MALDI Clinical database older than v6903	No ID
Vitek 2 version 8.01	*C. haemulonii* *C. duobushaemulonii* No ID/low discrimination
Vitek 2 version before 8.01	*C. haemulonii* *C. duobushaemulonii* *C. lusitaniae* *C. famata* No ID/low discrimination
API 20C AUX	*Rhodotorula glutinis* (characteristic red colour not present) *C. sake* No ID/low discrimination
API Candida	*C. famata*
BD Phoenix yeast identification system	*C. haemulonii* *C. catenulata* No ID

Abbreviations: *C., Candida*; MALDI, Matrix Assisted Laser Desorption Ionization; MS, mass spectrometry
